# Tamaractam, a New Bioactive Lactam from *Tamarix ramosissima*, Induces Apoptosis in Rheumatoid Arthritis Fibroblast-Like Synoviocytes

**DOI:** 10.3390/molecules22010096

**Published:** 2017-01-10

**Authors:** Yao Yao, Cheng-Shuai Jiang, Na Sun, Wei-Qi Li, Yang Niu, Huai-Qin Han, Zhen-Hua Miao, Xun-Xia Zhao, Jing Zhao, Juan Li

**Affiliations:** 1 School of Basic Medical Science, Ningxia Medical University, Yinchuan 750004, China; 15909511703@163.com (Y.Y.); hanhuaiqin76@sina.com (H.-Q.H.); miaozhh@yahoo.com (Z.-H.M.); zhaoxx@nxmu.edu.cn (X.-X.Z.); 2 School of Pharmacy, Ningxia Medical University, Yinchuan 750004, China; jiangcs1991@163.com (C.-S.J.); sunna_2012@sina.com (N.S.); 3 China National Center for Biotechnology Development, Beijing 100039, China; liwq1982@126.com; 4 School of Chinese Medicine, Ningxia Medical University, Yinchuan 750004, China; niuyangnxmu@sina.com; 5 Key Laboratory of Hui Medicine Modernization, Ministry of Education, Yinchuan 750004, China; 6 Tongji Hospital, Huazhong University of Science and Technology, Wuhan 430030, China; zhaojing1981@163.com; 7 Ningxia Engineering and Technology Research Center for Modernization of Hui Medicine, Yinchuan 750004, China

**Keywords:** *Tamarix ramosissima*, tamaractam, rheumatoid arthritis, fibroblast-like synoviocytes, growth inhibitory activity, apoptosis

## Abstract

Chemical investigation of *Tamarix ramosissima* Ledeb, a traditional herbal medicine used for rheumatoid arthritis (RA) treatment in northwest China, led to the discovery of a new phenolic aromatic rings substituted lactam, tamaractam (**1**), together with the previously reported compounds *cis*-*N*-feruloyl-3-*O*-methyldopamine (**2**) and *trans*-*N*-feruloyl-3-*O*-methyldopamine (**3**). The structures of the compounds were determined by high resolution electrospray ionization mass spectroscopy (HRESIMS) and 1D and 2D-NMR experiments, as well as comparison with the literature data. The effects of the three compounds on the viability of RA fibroblast-like synoviocytes (RA-FLS) were assessed by 3-(4,5-dimethyl-2-thiazolyl)-2,5-diphenyl-2-*H*-tetrazolium bromide (MTT) assay. Pro-apoptosis effect of compound **1** in RA-FLS was further investigated by terminal deoxynucleotidyl transferase-mediated dUTP nick-end labeling (TUNEL) assay, activated caspase-3/7 level assessment using luminescence assay, and sub-G_1_ fraction measurement using flow cytometry. It was found that these three compounds displayed variable proliferation inhibitory activity in RA-FLS, and compound **1** exhibited the strongest effect. Compound **1** could remarkably induce cellular apoptosis of RA-FLS, increase activated caspase-3/7 levels, and significantly increase sub-G_1_ fraction in the cell cycle. The results suggested that compound **1** may inhibit the proliferation of RA-FLS through apoptosis-inducing effect, and these compounds may contribute to the anti-RA effect of *T. ramosissima*.

## 1. Introduction

*Tamarix ramosissima*, commonly known as tamarisk or rose willow, is a shrub or dungarunga belonging to the family Tamaricaceae. The *Tamarix* species have strong adaptability to the arid desert environment and saline and alkaline soil. They are not only excellent sand fixing plants, but also a good species for soil and water conservation and afforestation in saline-alkali soil [1]. Various species of *Tamarix* have been used as herbal medicines in the treatment of inflammation, leucoderma, spleen troubles, and eye diseases [2]. Tender branches and leaves of *T. ramosissima* are one of the herbal medicines with a long history of use for the treatment of rheumatoid arthritis (RA) in northwest China, especially in the Ningxia Hui Autonomous Region. Previous pharmacological study revealed that this plant has antioxidant and antimicrobial activities [1]. In a chemical constituents study, this plant has been found to be rich in polyphenolic compounds such as flavonoids, phenolic acids, hydrolyzable tannins, and coumarins [3,4,5].

In our pursuit to find new, potential, natural anti-RA compounds, the chemical investigation of *T. ramosissima* was carried out. The study resulted in the identification of three structurally related compounds, including the new compound tamaractam (**1**) and the previously reported compounds *cis*-*N*-feruloyl-3-*O*-methyldopamine (**2**) and *trans*-*N*-feruloyl-3-*O*-methyldopamine (**3**). The anti-proliferation effects of the three compounds were assessed on RA fibroblast-like synoviocytes (RA-FLS), and the apoptosis-inducing effects of compound **1** were further investigated.

## 2. Results and Discussion

### 2.1. Purification of Compounds ***1**–**3***

**Figure 1:**
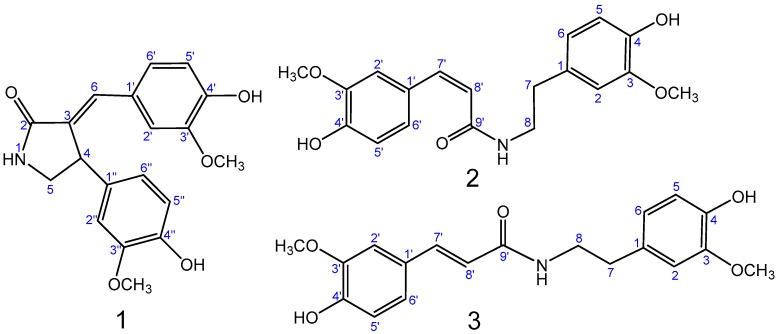
Structures of compounds **1**–**3**.

Samples of *T. ramosissima* were extracted with a mixture of MeOH/H_2_O (7:3). The crude extract was further extracted with petroleum ether; EtOAc and *n*-BuOH successively. The EtOAc extraction was subjected to chromatographic separation on normal SiO_2_, RP-18 columns, and prep-HPLC to provide one new compound, tamaractam (**1**), and two known compounds, *cis*-*N*-feruloyl-3-*O*-methyldopamine (**2**) and *trans*-*N*-feruloyl-3-*O*-methyldopamine (**3**) (Figure 1). The isolated compounds were evaluated for their anti-RA activity in vitro.

### 2.2. Structure Elucidation of Compounds ***1**–**3***

**Figure 2:**
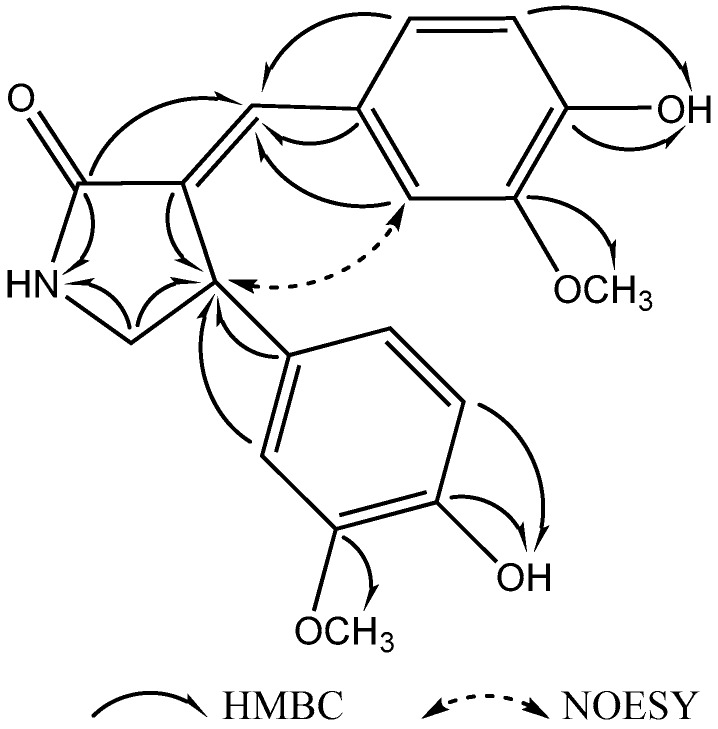
Selective HMBC and NOESY correlations of compound **1**.

Compound **1** (Figure 1) was obtained as a white amorphous powder, m.p. 249.4~250.3 °C. Its molecular formula was suggested as C_19_H_19_NO_5_ on the basis of the positive high resolution electrospray ionization mass spectroscopy (HRESIMS) pseudomolecular ion peak at *m*/*z* 342.1344 [M + H]^+^ and ^1^H- and ^13^C-NMR spectral analyses, requiring eleven degrees of unsaturation. The ^1^H-NMR spectrum of compound **1** (Table 1) displayed resonances for 19 protons, including two methoxyls (δ_H_ 3.51 and 3.67), one methylene (δ_H_ 3.78 and 3.03) and an adjacent methine (δ_H_ 4.53), six aromatic proton signals which belong to two ABX coupling systems (δ_H_ 6.66, 6.57, 6.83 and δ_H_ 6.68, 6.88, 6.86) [6], one enyl proton (δ_H_ 7.23), and three exchangeable singlet proton signals at δ_H_ 7.95, 8.84 and 9.34 for OH or NH moieties (Supplementary Materials Figure S1). The ^13^C-NMR spectrum (Table 1) showed signals for 19 carbons, including two methyls, one methylene, one methine, twelve aromatic carbons, two enyl carbons, and one carbonyl carbon (Figure S2). Analysis of the heteronuclear multiple-quantum correlation (HSQC) and the heteronuclear multiple bond correlation (HMBC) NMR spectrums (Figures S3–S5) led to the assembly of a double bond connected γ-butyrolactam skeleton by heteronuclear long range correlations between H-1(NH) at δ_H_ 7.95, the carbonyl carbon at δ_C_ 171.2 (C-2), and the methylene carbon at δ_C_ 49.1 (C-5); and correlations between H-4 at δ_H_ 4.53 and the enyl carbon at δ_C_ 131.1 (C-3), the methine carbon at δ_C_ 49.1 (C-5); as well as correlation between H-6 at δ_H_ 7.23 and the carbonyl carbon at δ_C_ 171.2 (C-2). Furthermore, two 4-hydroxy-3-methoxyphenyl moieties could be identified by the ABX coupling systems in the ^1^H-NMR spectrum, the correlations in HMBC spectrum between δ_H_ 3.51 (3′-OCH_3_) and the aromatic carbon at δ_C_ 147.4 (C-3′), between δ_H_ 3.67 (3′′-OCH_3_) and the aromatic carbon at δ_C_ 147.8 (C-3′′), in the correlations between δ_H_ 9.34 (4′-OH) and the aromatic carbon at δ_C_ 147.3 (C-4′), and between δ_H_ 8.84 (4′′-OH) and the aromatic carbon at δ_C_ 145.2 (C-4′′). The connection of these units was based on further analysis of the HMBC correlations (Figure 2). Correlations between H-4 at δ_H_ 4.53 and the aromatic carbons at δ_C_ 134.3 (C-1′′), 111.3 (C-2′′), and 118.7 (C-6′′) and correlations between H-6 at δ_H_ 7.23 and the aromatic carbons at δ_C_ 126.1 (C-1′), 112.9 (C-2′), and 124.5 (C-6′) indicated that these two 4-hydroxy-3-methoxyphenyl moieties were connected with the enyl γ-butyrolactam skeleton at C-4 and C-6.

The configuration of Δ^3,6^ double bond was detected and confirmed by NOESY correlations (Figure S6). The NOE between the H-4 at δ_H_ 4.53 and H-2′ at δ_H_ 6.86 indicated the *E*-configuration of the double bond (Figure 2). Moreover, the NOESY correlation between the H-4 and H-6 was not observed, which confirmed its *E*-configuration. Therefore, the structure of compound **1** was identified as (*E*)-3-(4′-Hydroxy-3′-methoxybenzylidene)-4-(4′′-hydroxy-3′′-methoxyphenyl) pyrrolidin-2-one (Figure 2). Compound **1** was generically named tamaractam.

The known compounds **2** and **3** (Figure 1) were identified by extensive analysis of their spectroscopic data, including ESIMS, ^1^H- and ^13^C-NMR data, as well as by comparison with the available data in the literature. Thus, the compounds were identified as *cis*-*N*-feruloyl-3-*O*-methyldopamine (**2**) [7] and *trans*-*N*-feruloyl-3-*O*-methyldopamine (**3**) [8].

### 2.3. In Vitro Anti-RA Activity of Compounds ***1**–**3***

Synovial hyperplasia is recognized as one of the key pathological characteristics of RA, which causes marginal bone erosion and joint destruction [9,10]. RA-FLS exhibit aggressive features, including hyperproliferation, apoptosis resistance, and high invasiveness [11,12]. Evidence suggested that RA-FLS play a pivotal role in the pathological process of synovitis and joint destruction in RA [13,14,15]. Therefore, RA-FLS were recruited for the investigation of anti-RA effect of compounds **1**–**3** in vitro.

**Figure 3:**
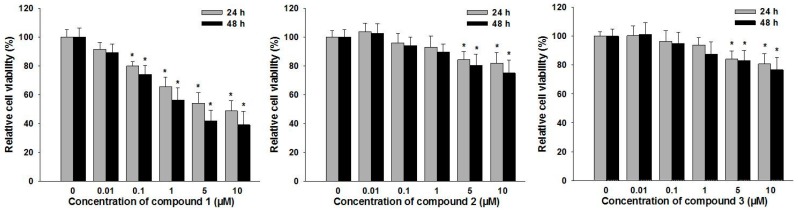
The effects of compounds **1**–**3** on RA-FLS cell viability. Cell viability was measured with 3-(4,5-dimethyl-2-thiazolyl)-2,5-diphenyl-2-*H*-tetrazolium bromide (MTT) assay after treatment with compounds **1**–**3** (0, 0.01, 0.1, 1, 5, and 10 μM) for 24 or 48 h. Data were shown as means ± S.E.M. of three independent experiments (* *p* < 0.05).

To evaluate the effects of the isolated compounds on cell viability, RA-FLS were incubated with various concentrations of compounds **1**–**3** (0.01, 0.1, 1, 5, and 10 μM) for 24 or 48 h, and then cell viability was assessed by 3-(4,5-dimethyl-2-thiazolyl)-2,5-diphenyl-2-*H*-tetrazolium bromide (MTT) assay. It is found that, compared with vehicle-treated control cells, 0.1~10 μM compound 1 treatment could significantly suppress the cell viability of RA-FLS in both time- and dose-dependent manners (*p* < 0.05); treatment with 0.01, 0.1, 1, 5 and 10 μM of compound 1 decreased cell survival by 91%, 80%, 65%, 54%, and 49%, respectively, after 24 h and by 89%, 74%, 56%, 42%, and 39% of the control viability level after 48 h, respectively (Figure 3). However, only 5 and 10 μM of compounds **2**–**3** treatment could induce significant suppression of cell viability (Figure 3). Based on these results, 0.1 and 1 μM of compound **1** were employed for subsequent apoptosis inducing experiments.

**Figure 4:**
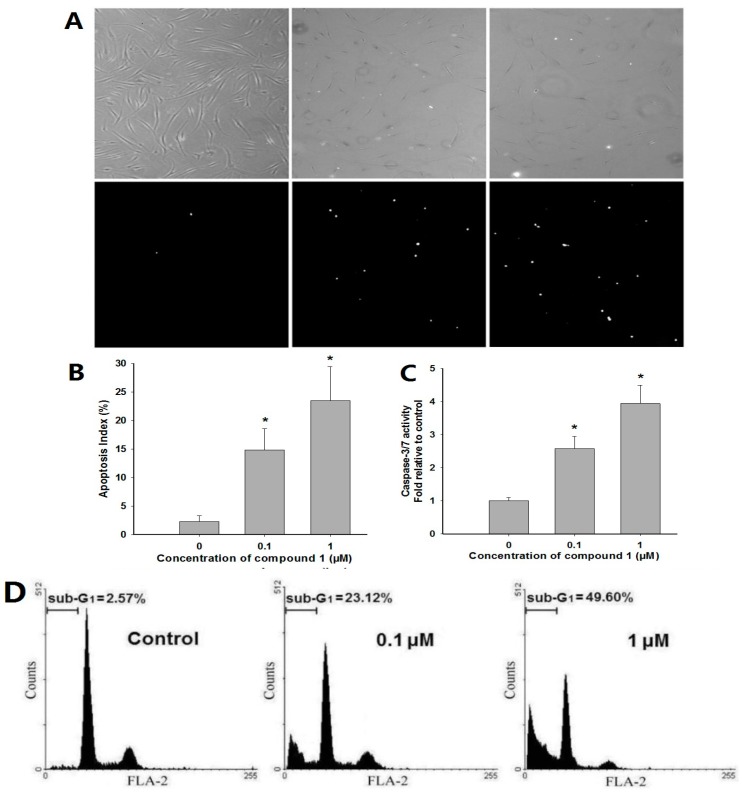
Effects of compound **1** on RA-FLS apoptosis. (**A**) After treatment with compound **1** (0.1 or 1 μM) or dimethyl sulphoxide (DMSO) vehicle for 48 h, apoptotic cell death measured by terminal deoxynucleotidyl transferase-mediated dUTP nick-end labeling (TUNEL) assay (×100). Bright spots in the lower panels are TUNEL-positive apoptotic cells. Corresponding phase contrast microscopy images are shown in the upper panels; (**B**) The apoptosis index of RA-FLS treated with 0.1 or 1 μM of compound **1** or DMSO vehicle. Data were shown as means ± S.E.M. of three independent experiments (* *p* < 0.05); (**C**) Effects of compound **1** on the levels of active caspase-3/7. RA-FLS cells were treated with 0.1 or 1 μM of compound **1** for 48 h and then processed to measure using the Caspase-Glo kit. Data were shown as means ± S.E.M. of three independent experiments (* *p* < 0.05); (**D**) Effects of compound **1** on sub-G_1_ fractions of RA-FLS. After treatment with compound **1** for 48 h at the concentration of 0.1 or 1 μM, cells were harvested by trypsinization, and analyzed using flow cytometry.

Terminal deoxynucleotidyl transferase-mediated dUTP nick-end labeling (TUNEL) assay was used to evaluate whether compound **1** treatment can influence apoptotic cell death of RA-FLS. It was found that treatment with 0.1 and 1 μM of compound **1** for 48 h significantly increased the number of TUNEL-positive cells compared to vehicle control cells (Figure 4A,B; 14.83% ± 3.76% and 23.52% ± 5.94%, respectively). Then the levels of activated caspase-3/7 were examined with a Caspase-Glo kit. It was found that, after 24 h incubation, compound **1** could significantly increase the levels of activated caspase-3/7 (Figure 4C; 0.1 and 1 μM are about 2.6- and 3.9-fold higher than untreated vehicle, respectively), indicating its potent pro-apoptotic effect [16]. Furthermore, the induction of apoptosis was confirmed by determination of sub-G_1_ fractions with a flow cytometric approach. After treatment with 0.1 or 1 μM of compound **1** for 48 h, cells were harvested and assessed by flow cytometry. The remarkably increased sub-G_1_ peak of apoptotic cells in the cell cycle indicated that compound **1** could potently induce apoptosis in RA-FLS (Figure 4D; for 0.1 and 1 μM, sub-G_1_ fractions were about 23.12% and 49.60%, respectively, while untreated vehicles represented 2.57%) [17].

Synovial hyperplasia is recognized as one of the major pathological characteristics of RA, which leads to marginal bony erosions and resultant joint destruction. RA-FLS play pivotal roles both in the initiation and the perpetuation of RA. These cells have been linked most prominently to the progressive destruction of articular cartilage [18]. There are several lines of evidence suggesting that rheumatoid synovia shows tumor-like expansion attributed to the resistance of RA-FLS to the apoptotic process [19,20]. Thus compounds with pro-apoptotic activity may provide a potent therapeutic approach for the treatment of RA. Our findings revealed that compound **1** had a remarkable apoptosis-inducing effect on RA-FLS in vitro. The in vivo anti-RA effect and the underlining mechanisms need further investigation.

## 3. Materials and Methods

### 3.1. General Experimental Procedures

Melting point was measured on a XT4 microscopic melting-point apparatus (Shanghai Jingke Instruments Company, Shanghai, China). Optical rotation was measured with a 241 polarimeter (Perkin-Elmer, Waltham, MA, USA). Electrospray ionization mass spectroscopy (ESIMS) data were recorded with a Micro TOF II spectrometer (Bruker, Bremen, Germany). High-resolution ESIMS data were recorded with an APEX II HR-TOF spectrometer (Bruker). NMR spectra were obtained in DMSO-*d*_6_ on a Bruker Avance DRX 400-MHz spectrometer at 400 MHz for ^1^H-NMR and 100 MHz for ^13^C-NMR. Precoated silica gel GF_254_ plates (Merck, Darmstadt, Germany) were used for TLC. For column chromatography, SiO_2_ (100–200 mesh, Qingdao Marine Chemical Factory, Qingdao, China) and Rp-C18 (ODS-A, 50 μm, YMC, Yantai, China) were used. HPLC purifications were performed on HPLC columns (YMC-Pack Pro C18, 5 μm, 250 mm × 4.6 mm and 250 mm × 10 mm, YMC, Kyoto, Japan) with a L-2000 HPLC system (Hitachi, Tokyo, Japan).

### 3.2. Biological Materials

Tender branches and leaves of *T. ramosissima* were collected from sandy land near Yellow River, Yinchuan, China. The samples were identified by Dr. Yunsheng Zhao, School of Pharmacy of Ningxia Medical University. A voucher sample was kept at Department of Medical Chemistry, School of Basic Medical Science, Ningxia Medical University under the registration code No. 2014050201.

### 3.3. Isolation and Purification of Compounds ***1**–**3***

The air-dried branches and leaves of *T. ramosissima* (5.0 kg) were smashed and extracted under refluxing condition with 70% EtOH/H_2_O (3 × 40 L). The successive extracts were combined and evaporated under reduced pressure to afford a crude extract (412 g), which was partitioned in 1 L H_2_O and extracted with petroleum ether (PE), EtOAc, and n-BuOH successively (4 × 1 L). Then these combined extracts were evaporated under reduced pressure separately to give three different crude extracts. The EtOAc extract (43 g) was subjected to a silica gel column with gradient elution (CH_2_Cl_2_-MeOH) to give 9 fractions (Fr.1 to Fr.9). Fr.6 (2.6 g), which was eluted with 10% MeOH in CH_2_Cl_2_, was fractionated on a Rp-C18 column using MeOH-H_2_O gradient eluent, affording 6 subfractions (Fr.6-1 to Fr.6-6). Fr.6-3 (110 mg) was further purified by recrystallization from MeOH to yield compound **1** (15 mg). Fr.4 (1.4 g), which was eluted with 5% MeOH in CH_2_Cl_2_, was fractionated on a silica gel column using PE-acetone gradient eluent, affording 5 subfractions (Fr.4-1 to Fr.4-5). Fr.4-5 (78 mg) was further purified on HPLC (YMC-Pack Pro C18, 5 μm, 250 mm × 10 mm, YMC, Kyoto, Japan), using 45% MeOH/H_2_O at a flow rate of 3.2 mL/min and UV detection at 220 nm, to yield compound **2** (7 mg) and **3** (18 mg).

### 3.4. Characterization of Compounds ***1**–**3***

*Tamaractam* (**1**): White crystal; m.p. 249.4~250.3 °C; ${\left[\alpha \right]}_{D}^{25}$ +15.6 (*c* 0.15, MeOH); HRESIMS *m*/*z* 342.1344 (calcd. for C_19_H_19_NO_5_, 342.1341 [M + H]^+^); NMR spectral data, see Table 1.

*Cis-N-feruloyl-3-O-methyldopamine* (**2**): White amorphous powder; ESIMS *m*/*z*: 344 [M + H]^+^; ^1^H-NMR (DMSO-*d*_6_, 400 MHz): δ_H_ 6.74 (1H, d, *J* = 2.0 Hz, H-2), 6.67 (1H, d, *J* = 8.0 Hz, H-5), 6.58 (1H, dd, *J* = 8.0, 2.0 Hz, H-6), 2.63 (2H, t, *J* = 7.6 Hz, H-7), 3.29 (2H, t, *J* = 7.6 Hz, H-8), 7.70 (1H, d, *J* = 2.0 Hz, H-2′), 6.71 (1H, d, *J* = 8.0 Hz, H-5′), 7.09 (1H, dd, *J* = 8.0, 2.0 Hz, H-6′), 6.48 (1H, d, *J* = 12.8 Hz, H-7′), 5.76 (1H, d, *J* = 12.8 Hz, H-8′), 3.71 (3H, s, 3-OCH_3_), 3.73 (3H, s, 3′-OCH_3_), 8.09 (1H, m, NH); ^13^C-NMR (DMSO-*d*_6_, 100 MHz): δ_C_ 130.2 (C-1), 112.7 (C-2), 147.4 (C-3), 144.8 (C-4), 115.3 (C-5), 120.7 (C-6), 34.7 (C-7), 40.5 (C-8), 126.7 (C-1′), 114.2 (C-2′), 146.8 (C-3′), 147.4 (C-4′), 114.9 (C-5′), 124.4 (C-6′), 136.8 (C-7′), 120.7 (C-8′), 166.2 (C-9′), 55.5 (2 × -OCH_3_).

*Trans-N-feruloyl-3-O-methyldopamine* (**3**): White amorphous powder; ESIMS *m*/*z*: 344 [M + H]^+^; ^1^H-NMR (DMSO-*d*_6_, 400 MHz): δ_H_ 6.78 (1H, d, *J* = 1.6 Hz, H-2), 6.69 (1H, d, *J* = 8.0 Hz, H-5), 6.60 (1H, dd, *J* = 8.0, 1.6 Hz, H-6), 2.66 (2H, t, *J* = 7.6 Hz, H-7), 3.37 (2H, t, *J* = 7.6 Hz, H-8), 7.11 (1H, d, *J* = 1.6 Hz, H-2′), 6.80 (1H, d, *J* = 8.0 Hz, H-5′), 6.98 (1H, dd, *J* = 8.0, 1.6 Hz, H-6′), 7.33 (1H, d, *J* = 15.6 Hz, H-7′), 6.45 (1H, d, *J* = 15.6 Hz, H-8′), 3.74 (3H, s, 3-OCH_3_), 3.79 (3H, s, 3′-OCH_3_), 7.99 (1H, m, NH); ^13^C-NMR (DMSO-*d*_6_, 100 MHz): δ_C_ 130.3 (C-1), 112.8 (C-2), 147.5 (C-3), 144.9 (C-4), 115.4 (C-5), 120.8 (C-6), 34.8 (C-7), 40.6 (C-8), 126.4 (C-1′), 110.8 (C-2′), 147.9 (C-3′), 148.4 (C-4′), 115.7 (C-5′), 121.6 (C-6′), 138.9 (C-7′), 119.0 (C-8′), 165.4 (C-9′), 55.5 (2 × -OCH_3_).

### 3.5. Biological Activity Assessment of Compounds ***1**–**3***

#### 3.5.1. RA-FLS Cell Culture

Human fibroblast-like synoviocytes from RA patients (RA-FLS) were purchased from Cell Applications, Inc., (San Diego, CA, USA) and cultured with a synoviocyte growth medium (Cell Applications). RA-FLS obtained from the 3rd to 5th passages were used for experiments. The study was conducted in accordance with the Declaration of Helsinki, and the protocol was approved by the Ethics Committee of Ningxia Medical University (Project identification code: 2014-036).

#### 3.5.2. Assessment of Cell Viability Using MTT Assay

Compounds **1**–**3** was dissolved in dimethyl sulfoxide (DMSO; Sigma-Aldrich Co., St. Louis, MO, USA). RA-FLS were seeded in 48-well plates at a density of 3 × 10^4^ cells/well, and were treated with 0.01, 0.1, 1, 5, and 10 μM of compounds **1**–**3** or DMSO vehicle only in a serum free medium. After 24 and 48 h of incubation, 2.5 mg/mL of 3-(4,5-dimethylthiazol-2-yl)-2,5-diphenyl-tetrazolium bromide (MTT) solution (Sigma-Aldrich) was added to the wells, and the cells were then incubated for 2 h. The absorbance of each well was measured by a Bio-Rad 680 microplate reader (Bio-Rad laboratories, Hercules, CA, USA) at 570 nm. Based on MTT assay results, compound **1** was employed in the subsequent apoptosis-inducing experiments.

#### 3.5.3. TUNEL Assay

Apoptosis in RA-FLS cells was measured by TUNEL assay (Roche Diagnostics, Mannheim, Germany), according to the manufacturer′s protocol. Briefly, RA-FLS were incubated with 0.1 and 1 μM of compound **1** for 48 h and then fixed with 4% paraformaldehyde for 30 min at room temperature. After washing with phosphate buffer saline (PBS), permeabilization solution (0.1% sodium citrate, 0.1% Triton X-100) was added for a 2 min reaction and cells were incubated with terminal deoxynucleotidyl transferase (TdT) and biotin-11-dUTP for 1 h at 37 °C. The nuclear morphology was observed by fluorescence microscopy IX71 (Olympus, Tokyo, Japan). The apoptosis index was calculated based on the percentage of TUNEL-positive cells in 1000 RA-FLS.

#### 3.5.4. Measurement of Caspase-3/7 Activity

The levels of activated caspase-3/7 in RA-FLS were further assessed with a Caspase-Glo kit (Promega, Madison, WI, USA) [21], according to the manufacturer’s protocol. Briefly, cells were plated at 1 × 10^4^ cells/well in 96-well plates and treated with 0.1 and 1 μM of compound **1** for 48 h. After incubation, cells were treated for 2 h with reconstituted Caspase 3/7-Glo reagent. Then the luminescence signal generated after cleavage of DEVD-aminoluciferin substrate by caspase 3/7 was detected using a luminometer plate reader (Luminoskan Ascent, Thermo Electron, Helsinki, Finland).

#### 3.5.5. Assessment of Sub-G_1_ Fraction

The induction of apoptosis was confirmed by a flow cytometric approach using propidium iodide (PI) as fluorescent stain [22]. Briefly, cells were plated in 24-well plates (1 × 10^5^ cells/well), in triplicate, and then treated with 0.1 and 1 μM of compound **1** for 48 h. Cells were harvested, washed twice with PBS, and the cellular DNA stained with 200 μL PI (50 μg/mL, RNase 1 μg/mL, Triton X-100 0.1%). After incubation at 4 °C for 20 min, the cells were analyzed by flow cytometry (FACS Calibur, BD Biosciences, San Jose, CA, USA) and sub-G_1_ fractions were computed using ModFit LT version 4.0 software (Verity Software House, Topsham, ME, USA).

### 3.6. Data Analysis

All statistical tests were performed using SPSS11.0 statistical software (SPSS, Chicago, IL, USA). The level of statistical significance was determined by using one-way analysis of variance (ANOVA). Results were expressed as the mean ± S.E.M. of three independent experiments performed in triplicate. Differences were considered significant when *p* < 0.05.

## 4. Conclusions

The investigation of the 70% EtOH/H_2_O extract of the traditional herbal medicine *T. ramosissima* yielded the new compound tamaractam (**1**), along with the previously reported *cis*-*N*-feruloyl-3-*O*-methyldopamine (**2**) and *trans*-*N*-feruloyl-3-*O*-methyldopamine (**3**). The structures of the isolated compounds were determined by HRESIMS and 1D and 2D-NMR data, as well as by comparison with the available data in the literature. These compounds displayed variable proliferation inhibitory activity, and compound **1** showed remarkable apoptosis-inducing effect on RA-FLS.

## Figures and Tables

**Table 1:** NMR spectral data of **1** (DMSO-*d*_6_, 400 and 100 MHz).

No.	δ_H_ (Mult., *J* (Hz))	δ_C_ (Mult.)
1	7.95 s	-
2	-	171.2 C
3	-	131.1 C
4	4.53 d (8.0)	41.9 CH
5	3.78 dd (10.0, 8.0)	49.1 CH_2_
	3.03 d (10.0)	
6	7.23 s	130.9 CH
1′	-	126.1 C
2′	6.86 d (2.0)	112.9 CH
3′	-	147.4 C
4′	-	147.3 C
5′	6.68 d (8.4)	115.3 CH
6′	6.88 dd (8.4, 2.0)	124.5 CH
1′′	-	134.3 C
2′′	6.83 d (2.0)	111.3 CH
3′′	-	147.8 C
4′′	-	145.2 C
5′′	6.66 d (8.4)	115.6 CH
6′′	6.57 dd (8.4, 2.0)	118.7 CH
3′-OCH_3_	3.51 s	55.2 CH_3_
3′′-OCH_3_	3.67 s	55.5 CH_3_
4′-OH	9.34 s	-
4′′-OH	8.84 s	-

## Supplementary Materials

Supplementary materials can be accessed at: http://www.mdpi.com/1420-3049/22/1/96/s1.
