# Supplementary material for: Tamaractam, a New Bioactive Lactam from Tamarix ramosissima, Induces Apoptosis in Rheumatoid Arthritis Fibroblast-Like Synoviocytes
Source: Molecules. 2017 Jan 10;22(1):96. doi: 10.3390/molecules22010096 (PMC6155678; doi:10.3390/molecules22010096)
Supplement: Supplementary file 1 [file molecules-22-00096-s001.pdf]

Supplementary Materials: Tamaractam, a New Bioactive Lactam from *Tamarix ramosissima* Induces Apoptosis in Rheumatoid Arthritis Fibroblast-Like Synoviocytes

Yao Yao, Cheng-Shuai Jiang, Na Sun, Wei-Qi Li, Yang Niu, Huai-Qin Han, Zhen-Hua Miao, Xun-Xia Zhao, Jing Zhao and Juan Li

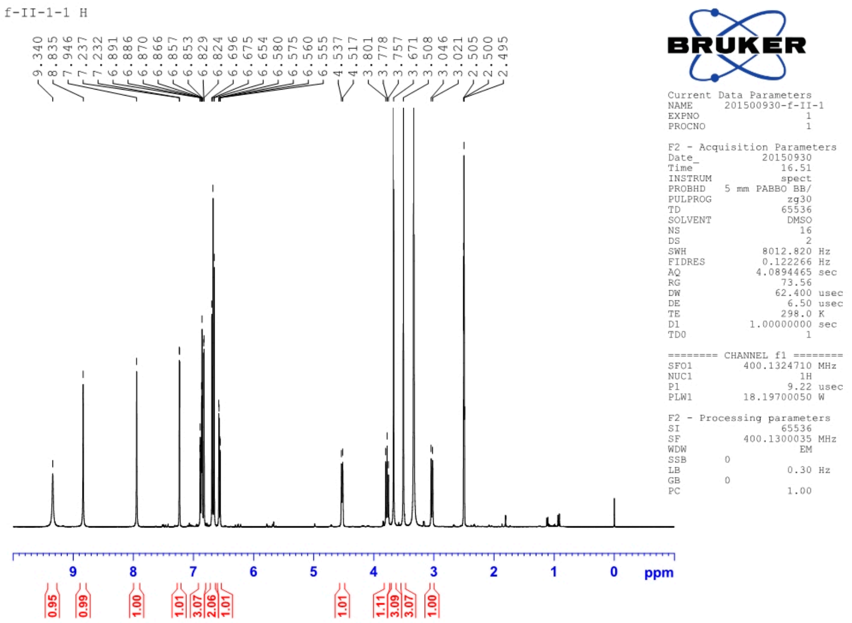

Figure S1. Tamaractam-^{1}H.

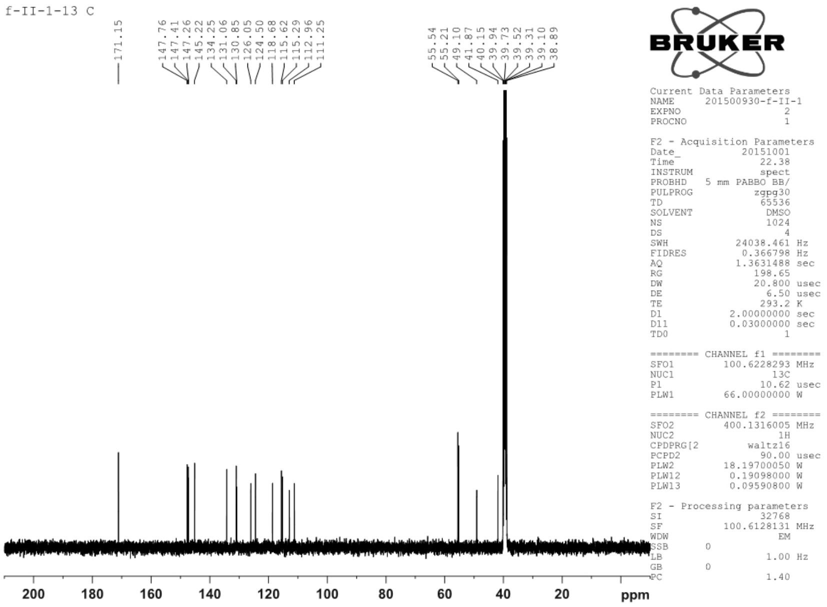

Figure S2. Tamaractam-^{13}C.

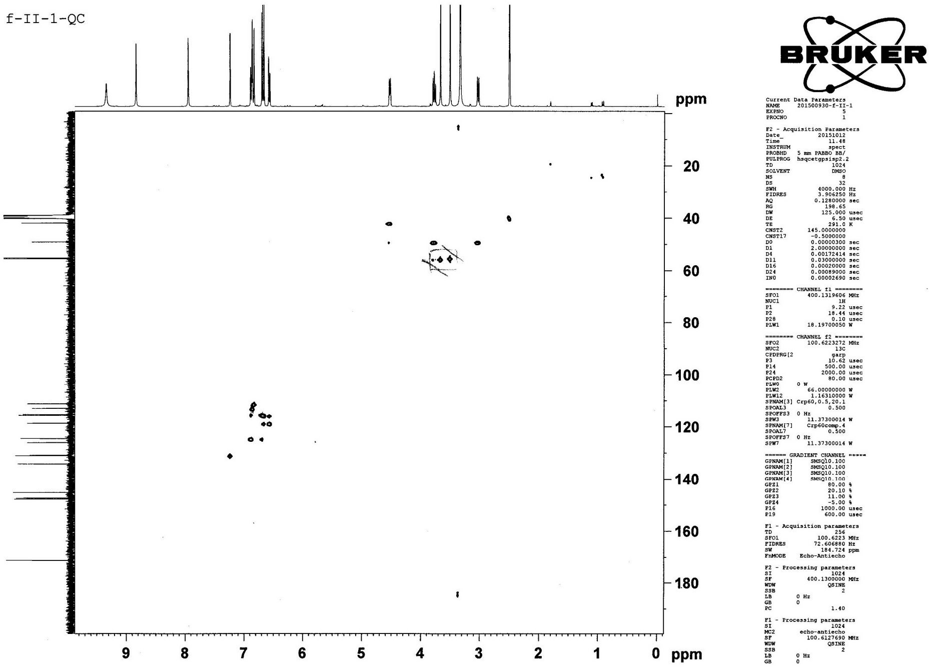

Figure S3. Tamaractam-QC.

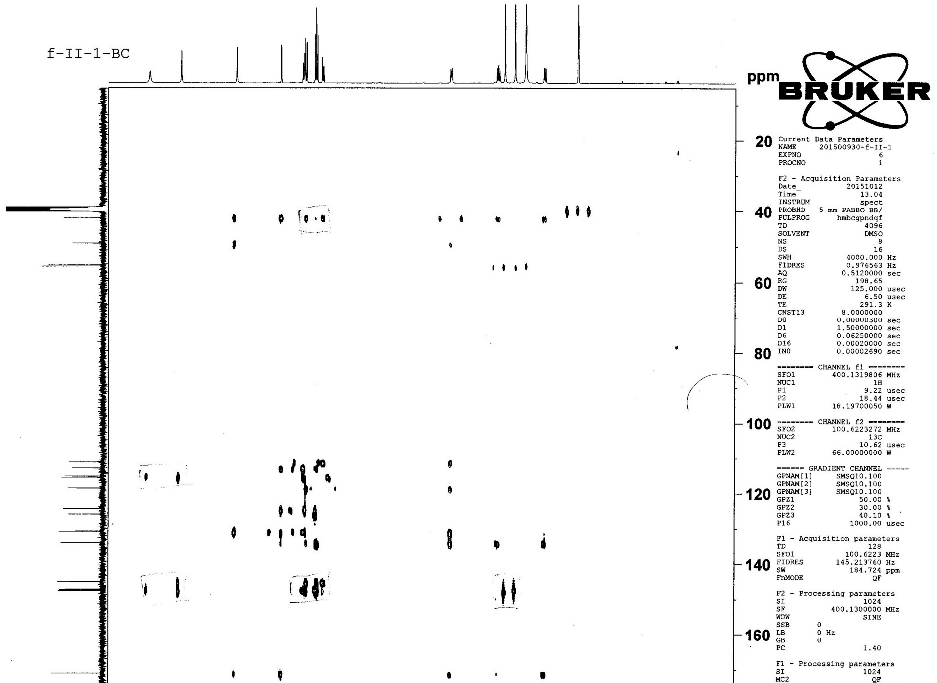

Figure S4. Tamaractam-BC1.

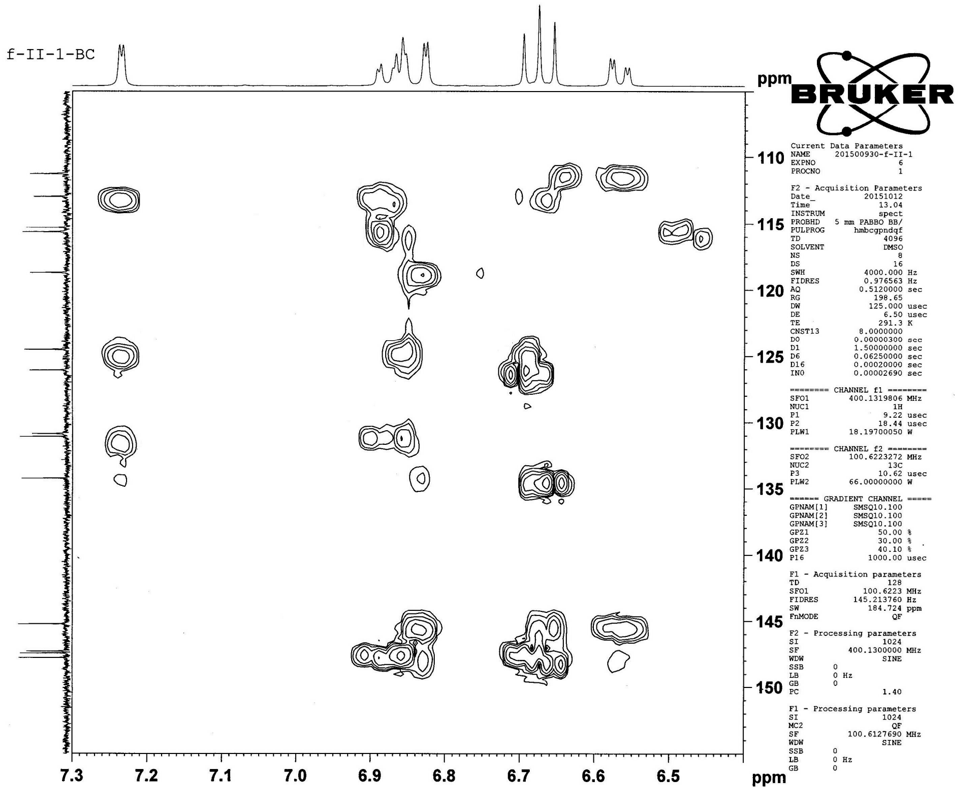

Figure S5. Tamaractam-BC2.

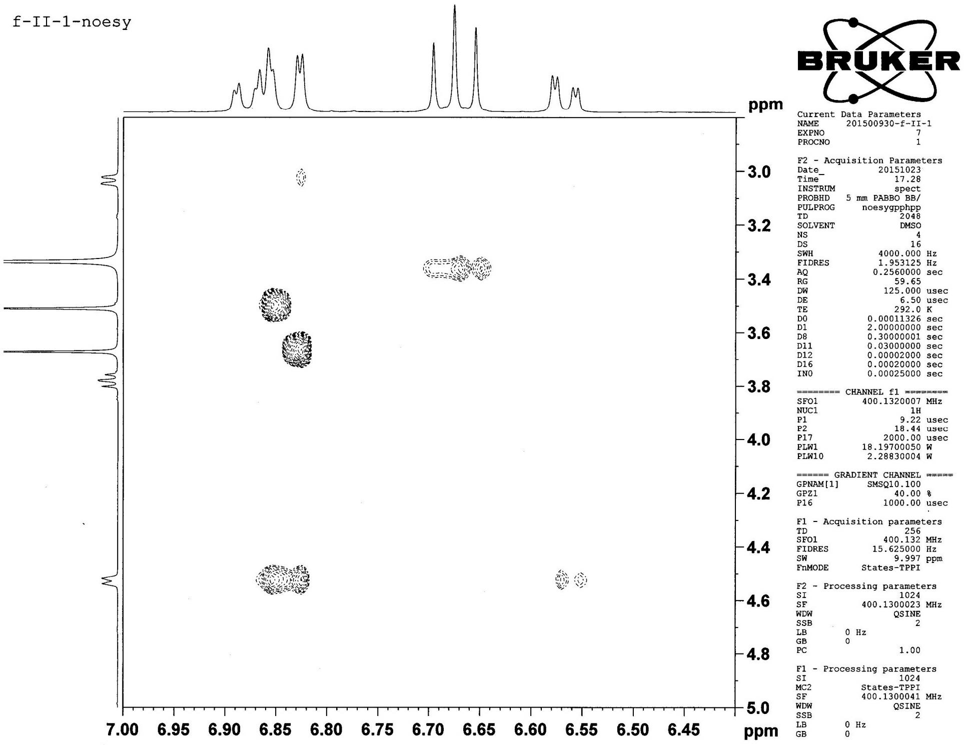

Figure S6. Tamaractam-noesy.
